# Can Heart Valve Decellularization Be Standardized? A Review of the Parameters Used for the Quality Control of Decellularization Processes

**DOI:** 10.3389/fbioe.2022.830899

**Published:** 2022-02-17

**Authors:** F. Naso, A. Gandaglia

**Affiliations:** Biocompatibility Innovation Srl, Este (PD), Italy

**Keywords:** biocompatibility, heart valve decellularization, protocols standardization, xenoantigens removal, regenerative medicine

## Abstract

When a tissue or an organ is considered, the attention inevitably falls on the complex and delicate mechanisms regulating the correct interaction of billions of cells that populate it. However, the most critical component for the functionality of specific tissue or organ is not the cell, but the cell-secreted three-dimensional structure known as the extracellular matrix (ECM). Without the presence of an adequate ECM, there would be no optimal support and stimuli for the cellular component to replicate, communicate and interact properly, thus compromising cell dynamics and behaviour and contributing to the loss of tissue-specific cellular phenotype and functions. The limitations of the current bioprosthetic implantable medical devices have led researchers to explore tissue engineering constructs, predominantly using animal tissues as a potentially unlimited source of materials. The high homology of the protein sequences that compose the mammalian ECM, can be exploited to convert a soft animal tissue into a human autologous functional and long-lasting prosthesis ensuring the viability of the cells and maintaining the proper biomechanical function. Decellularization has been shown to be a highly promising technique to generate tissue-specific ECM-derived products for multiple applications, although it might comprise very complex processes that involve the simultaneous use of chemical, biochemical, physical and enzymatic protocols. Several different approaches have been reported in the literature for the treatment of bone, cartilage, adipose, dermal, neural and cardiovascular tissues, as well as skeletal muscle, tendons and gastrointestinal tract matrices. However, most of these reports refer to experimental data. This paper reviews the most common and latest decellularization approaches that have been adopted in cardiovascular tissue engineering. The efficacy of cells removal was specifically reviewed and discussed, together with the parameters that could be used as quality control markers for the evaluation of the effectiveness of decellularization and tissue biocompatibility. The purpose was to provide a panel of parameters that can be shared and taken into consideration by the scientific community to achieve more efficient, comparable, and reliable experimental research results and a faster technology transfer to the market.

## Introduction

Generally, in biological tissue, the main component in terms of volume is not related to the cells, but rather to the cell-secreted three-dimensional extracellular matrix (ECM; Table 1). The ECM provides structural and histoarchitectural integrity and mechanical support in the tissues and organs, working actively in the exchange of ions, nutrients, waters, metabolites, and signals (Gattazzo et al., 2014). The sequencing of the human genome revealed hundreds of proteins that are involved in the constitution of the mature ECM (Naba et al., 2011). The set of these proteins constitutes the matrisome that comprises approximately 1–1.5% of the mammalian proteome (Hynes and Naba, 2011). The ECM acts as a multi-functional environment in which tissue-resident cells attach, communicate and interact, thereby regulating cell dynamics and behaviour and contributing to the maintenance of tissue-specific cell phenotypes and functions. To date, a number of different animal-tissue-derived ECMs have been used to produce bioprosthetic (chemically treated) substitutes for various applications such as for bone, cartilage, muscle, tendon, vascular graft, heart valve, nerve, dermal and gastrointestinal tract tissue repair or replacement (Badylak et al., 2009; Brown and Badylack, 2014; Folli et al., 2018). Many of these biological medical devices have been subjected to treatments that allow preservation of their functionality, but not the viability of their cellular content. Non-viable tissue is not capable of ECM regeneration and remodelling, thus limiting their lifespan and imposing the need for frequent replacement, forcing patients to multiple surgical interventions. To overcome this limitation and develop viable and functional engineered animal-derived ECMs, native tissues have been subjected to controlled removal of their cellular content, generating a decellularized three-dimensional scaffold (Crapo et al., 2011). Reported decellularization protocols have included a combination of physical techniques, detergents, enzymes and chemical compounds that have been shown not to adversely affect the architectural, ultrastructural, mechanical or biological integrity of the ECM (see Table 2). Unfortunately, there has been a lack of standardized assessment of tissue-specific decellularization methods, which compromises the effective comparison of the efficiency of different protocols (Bruyneel and Carr, 2017). Post-decellularization tissue characterization has included *in vitro* and *in vivo* studies providing data that are limited to the removal of the endogenous DNA in the ECM scaffolds and the safety of implantable commercial products. However, even when marketed decellularized products have been certified with the ISO standard for biological medical devices, several adverse reactions have been reported when introduced into the clinical setting (Simon et al., 2003; Kasimir et al., 2005; Ruffer et al., 2010; Woo et al., 2016). In particular, clinical application of decellularized porcine pulmonary valves exhibited massive inflammatory reaction associated with necrosis and graft stenosis in adult patients (Breitenbach et al., 2014—; Perri et al., 2012) with a relevant percentage of patients needed reoperation. Similarly, porcine intestinal submucosae (SIS) adopted for heart valve repair and reconstruction exhibited the presence of inflammatory cells, fibrosis and calcification after 4 years from the implant (Padalino et al., 2016—Hofmann et al., 2017—Mosala et al., 2017).

**TABLE 1:** Description of the most common components that contribute to forming the ECM.

ECM component	Features and function	
**COLLAGEN**	Representing the structural proteins that are the most abundant in the ECM. The molecular feature of collagen is the glycine–*X*–*Y* triplet repeat, where *X* frequently represents proline and *Y* represents hydroxyproline. Collagen provides structural strength to the ECM.	**Type I**
		Fibrillar collagen, primarily found in the ECM of skin and bone. Type I collagen is by far the most abundant type of all collagens. Genetic defects can cause osteogenesis imperfecta.
		**Type II**
		Fibrillar collagen, primarily found in the ECM of cartilage.
		**Type III**
		Fibrillar collagen, primarily found in the ECM of elastic tissues such as lung and blood vessels.
		**Type IV**
		Network-forming collagen, primarily found in the basement membrane.
		**Type V**
		Fibrillar collagen found in the ECM of a wide variety of different tissues.
**ELASTIN**	Mature elastin proteins result in a coiled conformation that allows these proteins to expand and contract in response to tensile stress. Elastin thus endows the ECM with elastic recoil and is abundant in tissues that require frequent expansion and contraction. Predominant tissues requiring this function are the lungs and cardiovascular ones.	
**PROTEOGLYCANS**	Proteoglycans represent a diverse group of ECM core proteins that are covalently bound to glycosaminoglycans (GAGs). GAGs are unbranched carbohydrates that consist of repeating disaccharide subunits that vary in number, these saccharide elements can undergo modification by epimerization and sulfation resulting in a vast diversity of GAG chains. Through these GAG chains, proteoglycans acquire a high negative charge leading to the attraction of positively charged electrolytes and the movement of water molecules that travel by osmosis to the proteoglycan. The resulting collection of molecules forms a gel that can expand and fulfil important ECM hydration and lubrication effects	**Heparan sulfate**
		Heparan sulfate (HS) is a linear polysaccharide found in all animal tissues. It occurs as a proteoglycan (PG) in which two or three HS chains are attached near to the cell surface or ECM proteins. It is in this form that HS binds to a variety of protein ligands and regulates a wide variety of biological activities, including developmental processes, angiogenesis, blood coagulation, and tumour metastasis. In the extracellular matrix, especially basement membranes, the multi-domain proteins perlecan, agrin, and collagen XVIII are the main proteins to which heparan sulfate is attached.
		**Chondroitin sulfate**
		Chondroitin sulfates (CS) contribute to the tensile strength of cartilage, tendons, ligaments, and walls of the aorta. They have also been known to affect neuroplasticity.
		**Keratan sulfate**
		Keratan sulfates (KS) have a variable sulfate content and, unlike many other GAGs, do not contain uronic acid. They are present in the cornea, cartilage, bones, and horns of animals.
**HYALURONIC ACID**	Non-proteoglycan polysaccharide hyaluronic acid (or “hyaluronan”) is a polysaccharide consisting of alternating residues of D-glucuronic acid and N-acetylglucosamine, and unlike other GAGs, is not found as a proteoglycan. Hyaluronic acid in the extracellular space confers upon tissues the ability to resist compression by providing a counteracting turgor (swelling) force by absorbing significant amounts of water. Hyaluronic acid is thus found in abundance in the ECM of load-bearing joints. It is also a chief component of the interstitial gel. Hyaluronic acid is found on the inner surface of the cell membrane and is translocated out of the cell during biosynthesis. Hyaluronic acid acts as an environmental cue that regulates cell behaviour during embryonic development, healing processes, inflammation, and tumour development. It interacts with a specific transmembrane receptor, CD44.	
**GLYCOPROTEINS**	Glycoproteins greatly contribute to making the ECM a cohesive network of molecules, although they also perform other functions. Glycoproteins are intermediaries that link structural molecules between each other, and also link structural molecules and cells. In each glycoprotein molecule, several domains are binding different molecules that altogether form cross-linked molecular networks. Fibronectins, laminins and tenascins are major glycoproteins of the animal ECM.	**Fibronectin**
		Fibronectin is a dimer comprised of two similar monomers that have a molecular weight of 220–250 kDa and are covalently linked on the C-terminal ends. Each monomer contains repeating motifs that are organized into distinct functional domains. These domains allow the binding of specific ligands, like integrins and collagens, that participate in signal transduction and contribute to the regulation of cellular activities such as branching morphogenesis. Fibronectins also help at the site of tissue injury by binding to platelets during blood clotting and facilitating cell movement to the affected area during wound healing.
		**Laminin**
		Laminins are proteins found in the basal laminae of virtually all animals. Rather than forming collagen-like fibers, laminins form networks of web-like structures that resist tensile forces in the basal lamina. They also assist in cell adhesion. Laminins bind other ECM components such as collagens and nidogens.
		**Tenascins**
		The molecular structure shows a modular hexameric organization. Several isoforms of tenascin can be obtained by alternative splicing of the messenger RNA. Tenascin-C is released into the extracellular matrix of tendons, bones and cartilage during embryonary development. Tenascin-C is overexpressed as a consequence of tissue damages like a heart attack. Tenascin-R is abundant in the nervous system, both during development and in adults. Tenascin-X is present in the connective tissue and can be abundant in muscles under heavy activity, like in professional athletes. Like other glycoproteins, tenascins change the cohesive state of the ECM by binding integrins, fibronectins, collagens and proteoglycans. In animals, each type of tenascin is expressed in particular locations of the organism, that may change during development. The expression of tenascins is induced in tissues being repaired, or during tumour and pathological processes.

**FIGURE 1:**
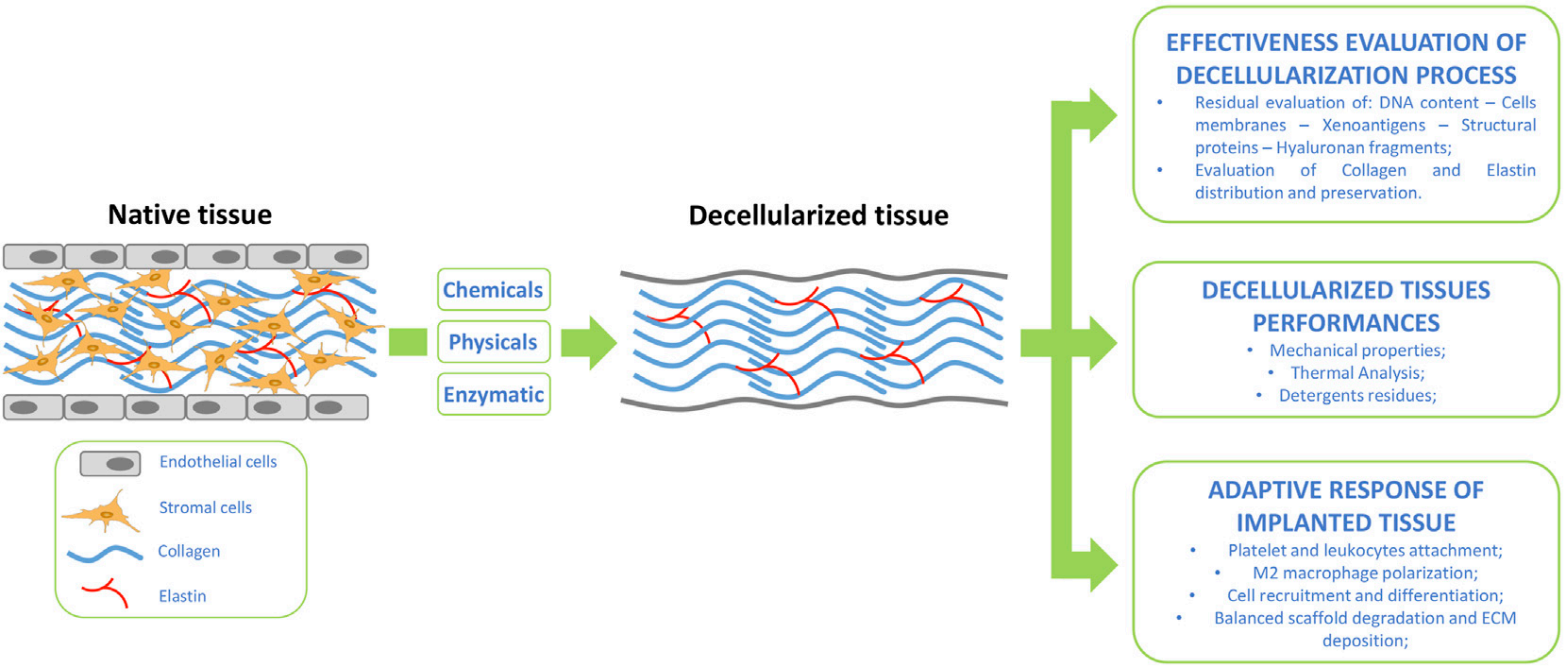
Tissue decellularization flow-scheme supported by suggested assessment for post tissue treatment evaluation and implanted graft integration.

**TABLE 2:** The most common decellularization approaches.

Decellularization approaches	Type		Rationale	Collateral effects	References
**CHEMICALS**	Acidic and alkaline solutions	Acetic-, peracetic-, hydrochloric- and sulfuric-acid as well as ammonium hydroxide	Disruption of cell membranes and intracellular organelles	Very aggressive toward the ECM protein such as collagen and to GAGs	Gilbert et al., (2006)
					Gilbert et al., (2005)
					Reing et al., (2010)
					Cornelison et al., (2018)
	Detergents	Non-ionic (Triton X-100), anionic (SDS, SDC), zwitterionic (CHAPS)	Solubilization of cell membranes and lipids	Possibility of denaturing proteins if used for a long time, difficulty of removal, and limited permeability	Korossis et al. (2002)
					Naso and Gandaglia, (2018)
					Wilson et al., (1995)
					Konertz et al., (2005) Erdbrügger et al., (2006)
					Quint et al. (2011)
					Mendibil et al. (2020)
	Protease inhibitors	Phenylmethylsulfonylfluoride, aprotinin and leupeptin	Avoidance of ECM damages by endogenous proteases leaked from the lysed cells	Difficulty of removal, residual toxicity for cellular repopulation	Luo et al. (2014)
					Gallo et al., (2012)
	Antibiotics	Penicillin, streptomycin, or amphotericin B	Prevention of contamination	n.d	Rajab et al., (2020)
					Gilpin and Yang, (2017)
					Lin et al., (2004)
					Leyh et al., (2003)
**ENZYMATIC**	Nucleases	Endo- or exo-nucleases	Degradation of nucleic acids	n.d	Haupt et al. (2018)
	Proteases	Trypsin, pepsin, protease K	Proteins degradation	Alteration of the ECM structure, degradation of laminin, and removal of GAGs, resulting in severe mechanical weakness of the treated tissue	Naso and Gandaglia, (2018)
					Rieder et al., (2004)
					Poon et al. (2013)
	Lipases	Pancreatic, gastric lipase	Lipids hydrolysis and solubilization	n.d	Gilbert, (2012)
					Schenke-Layland et al. (2003)
	Di-saccharidases	alpha1,3 galactosidase, N-glycolylneuroaminidase	Removal of alpha-Gal and Neu5Gc xenogeneic epitopes	n.d	Gilbert, (2012)
					Naso et al., (2017)
**PHYSICALS**	Freeze-thawing	n.d	Mostly combined with shaking or agitation. Cell lysis is caused by the formation of ice crystals following the freezing process	Damaging of the ECM, cellular debris can persist on the ECM	Toumpaniari et al., (2020)
					Curtil et al., (1997a), Curtil et al., (1997b)
					Hung et al., (2013)
					Nonaka et al., (2013)
					Waletzko et al., (2020)
					Butler et al., (2017)
	Mechanical force	n.d	Facilitating chemical agent infiltration to achieve better cell lysis (shaking, agitation, high hydrostatic pressure, supercritical carbon dioxide)	Avoiding tissues with fragile ECM.	
	Perfusion (vacuum-assisted)	n.d	Endogenous vascular catheters provide to delivering decellularization solution within tissue even with the high structural organization	It is not always possible to apply, it is susceptible to bacterial infections and air embolization	

In addition, the literature survey has indicated that many different parameters have not been carefully considered in the assessment of decellularised scaffolds, even though they have been shown to be related to adverse events and/or dramatic side effects that compromised the functionality of the prosthesis in the patient and, ultimately, the patient’s health.

## Heart Valve Decellularization

Each year, more than 450.000 patients undergo heart valve replacement worldwide, and this number is estimated to reach 1.000.000 by 2050 due to the ageing population and the westernization of the lifestyle in several areas of the world, towards unbalanced diets with fatty foods and, consequently, the increase in cardiovascular disease (Naso and Gandaglia, 2018). Therapies for heart valve disease have most commonly comprised interventional and surgical approaches since pharmacological agents only address symptoms management. Surgical approach to dysfunctional heart valves can be addressed through the use of biological or mechanical prostheses. Bioprosthetic heart valves (BHVs) fabricated from bovine, porcine, or equine pericardium, bovine jugular vein, or porcine heart valve leaflets feature low thrombogenicity with no need for lifelong anti-coagulants (Carpentier et al., 1969). Unfortunately, the longevity of BHVs is limited by structural valve degeneration (SVD), which begins within 5 years after the implantation and typically necessitates BHV replacement after 10–12 years from the surgery (Shinkawa et al., 2015). In particular, BHVs substitutes are subjected to structural degradation, calcification and immune response (Koziarz et al., 2020) as a consequence of incomplete biocompatibility of the animal tissue (frequently bovine pericardium) adopted for their manufacture (Simionescu et al., 2021). In fact, the treatment with glutaraldehyde (GLU, considered as the standard method to ensure tissue biocompatibility, increased mechanical strength, sterilization and safe storage of BHVs) is not able to reach complete masking of the antigenic determinants of animal origin (Naso et al., 2013a). The *in vivo* interaction between such antigens and the patient’s circulating antibody was shown to exert a powerful pro-calcific effect. Moreover, GLU is responsible be, by itself, a potential calcium-binding site due to the presence of residual aldehydes, acids and Schiff bases.

Evidence suggests that also homograft (allogeneic valves) suffers from a loss of function due to early donor-specific antibody and T cell-mediated responses to human leukocyte antigens (MHC Class 2 HLA-DR) inducing fever and vascular disfunction (Dignan et al., 2000). In particular, concerns have been expressed about the durability of homograft valves in younger pediatric patients after the failure within 8 months in a small clinical retrospective study, due to T- and B-lymphocytic infiltrates (Rajani et al., 1998).

Mechanical valves (tilting disk or bi-leaflets) are made from metal and/or pyrolytic carbon. The main disadvantage of this type of valve is the formation of thrombotic clots in the stagnation points and/or hemolysis which requires lifelong anticoagulation therapy for the patients (Harris et al., 2015).

Notwithstanding the tremendous technological progress in the field of heart valve therapy, an ideal heart valve replacement has yet to be developed, with current replacement options lowering the quality of life and the life expectancy of heart valve replacement patients when compared to age-matched healthy individuals (Huygens et al., 2016). In addition, heart valve replacements are very costly (circa 15 billion Euros annually in the United States and Europe), mainly due to the postoperative care costs and the need for re-operations (Huygens et al., 2018; Etnel et al., 2019). A clear, unmet clinical need remains for a heart valve substitute that does not degenerate, can adjust to functional and somatic changes, and can be implanted via minimally invasive techniques. To overcome these limitations the xenogeneic decellularised heart valve has been considered as an attractive alternative for the development of a viable biological valve, capable of remodelling and regeneration.

## Decellularization Techniques

### Chemicals Approaches

A variety of chemicals, including detergents, solvents, acidic and alkaline solutions, and ionic solutions have been used to solubilize cellular membranes along with mechanical agitation or perfusion. Alkaline and acid treatments are very effective in solubilizing the cytoplasmic components; however, they have been rarely used since they are very aggressive towards the ECM proteins. Acetic-, peracetic-, hydrochloric- and sulfuric-acid, as well as ammonium hydroxide, are some examples of chemicals that have been shown to disrupt cellular membranes and intracellular organelles, whilst also dissociating important molecules, such as collagen, protein-protein bonds and GAGs (Gilbert et al., 2005; Gilbert et al., 2006; Reing et al., 2010; Cornelison et al., 2018).

Detergents have been used to solubilize cellular membranes and dissociate the intracellular structure. Nonionic detergents, such as Triton X-100 have been largely used in several decellularization protocols (Malone et al., 1984; Wilson et al., 1995; Bader et al., 1998; Kim et al., 2002; Meyer et al., 2005; Gallo et al., 2012), and has been shown to target the lipid–lipid and lipid-protein chemical bonds, leaving unaltered protein-protein interactions (Cartmell and Dunn, 2000; Woods and Gratzer, 2005). Although Triton X-100 is effective in the decellularization of tissues in which the key ECM component is primarily proteins, it has been generally avoided for the decellularization of tissues particularly rich in glycosaminoglycans (GAGs) (Korossis et al., 2002—; Booth et al., 2002). Moreover, Triton X-100 has often been adopted to remove the remnants of anionic detergents (sodium dodecyl sulfate; SDS) (Naso and Gandaglia, 2018). Anionic detergents including sodium deoxycholate (SDC) and SDS have been also used in many reported studies (Wilson et al., 1995; Korossis et al., 2002; Konertz et al., 2005; Erdbrügger et al., 2006; Gallo et al., 2012). SDS is reported to solubilize both the external and nuclear membranes, but it has also been recognized to denature proteins, altering the native structure of the ECM (Chen et al., 2004; Elder et al., 2010). Owing to this, short treatments with SDS could be a reasonable choice, aiming at minimizing the possible damage to the ECM proteins and overall structure (Kim et al., 2002 -; Tavassoli et al., 2015). Nevertheless, SDS is considered very proficient in removing nuclear and cytoplasmic waste, even though it is more challenging to be rinsed out of the tissue due to its ionic nature (Rajab et al., 2020—; Syed et al., 2014—; Dettin et al., 2017).

Zwitterionic detergents share features of both nonionic and ionic detergents. They are electrically neutral molecules that have both positive and negative charges. The tendency of these detergents to denature proteins has been demonstrated to be lower than ionic detergents, but higher than nonionic ones. CHAPS {3-[(3-cholamidopropyl)dimethylammonio]-1-propanesulfonate} has been a commonly used zwitterionic detergent (Quint et al., 2011). Noteworthy, zwitterionic detergents have been reported not to be effective permeating agents and, therefore, have been mainly used to decellularize thin tissue (Mendibil et al., 2020).

During decellularization, endogenous proteases have been reported to leak in high amounts from the lysed cells, risking irreversible damage to the ECM (Crapo et al., 2011). Phenylmethylsulfonylfluoride (PMSF), aprotinin and leupeptin are common protease inhibitors that have been adopted to prevent proteolysis (Gallo et al., 2012; Luo et al., 2014). Some decellularization protocols have also included antibiotics, such as penicillin, streptomycin, or amphotericin B, to prevent tissue contamination during treatment (Leyh et al., 2003; Lin et al., 2004; Gilpin and Yang, 2017; Rajab et al., 2020).

### Enzymatic Approaches

Biological protocols for tissue decellularization have involved enzymatic reactions, mainly to eliminate cell debris and other undesirable components of the ECM such as nucleic acids residues, fragments of cell membranes and mitochondrial DNA (Londono et al., 2017; Naso and Gandaglia, 2018). The removal of the nucleic acids residues has been of paramount importance in all decellularization processes, due to their tendency to remain stuck to ECM proteins and attract circulating calcium salt. To this end, decellularization protocols have utilized nucleases to catalyze the degradation of nucleic acids. Nucleases have been generally classified into endo- and exo-nucleases that cleave phosphodiester bonds within the nucleic acids and nucleotides from the end of nucleic acids respectively (Haupt et al., 2018).

Proteases have been used to catalyze the degradation of proteins and the effective detachment of the cellular component from its connective support. Among them, trypsin and pepsin have been the most commonly used proteases in decellularization protocols (Naso and Gandaglia, 2018), with the formers targeting the C-side bonds in arginine and lysine, and the latter being a highly aggressive protease commonly found in the stomach and also targeting the bonds between peptides. Noteworthy, the prolonged exposure to proteolytic enzymes has been reported to significantly alter the structure of the ECM, destroying laminin and removing GAGs, thus resulting in severe mechanical weakness of the tissue scaffold (Rieder et al., 2004; Poon et al., 2013; Naso and Gandaglia, 2018).

With a view to removing additional tissue components, different enzymes such as lipase that hydrolyzes lipids and can be useful for fatty tissues, and α-galactosidase that has been shown to be effective in removing the galactose-α-(1,3)-galactose xenoepitope, responsible for xenorejection in humans (Gilbert, 2012) have been used. Due to the aggressiveness of the enzymatic treatments on the ECM proteins, the concentration of the enzymes as well as the duration of the enzymatic treatment, have been shown to be critical parameters for achieving maximal decellularization effectiveness and minimal ECM degradation (Schenke-Layland et al., 2003).

### Physical Approaches

Physical techniques alone have been shown inadequate to produce effective tissue decellularization. As such, they have been used in combination with chemical and/or enzymatic processes. Physical methods that have been reported to facilitate the decellularization process are summarized in Table 2. Briefly, freezing-thawing, direct pressure, agitation, sonication, vascular perfusion, thermal shock, ultrasonic and manual disruption have been shown to facilitate cell disruption, transportation of the decellularization solution to cells and washing out of the cellular debris from tissues (Toumpaniari et al., 2020).

### Programmed Cell Death Approach

Studies have shown that suitable chemical signals can be coordinated for an intentional stimulation of the endogenous cellular apoptotic pathway. This approach has been reported to completely depend on the distribution of the precise ligands (Tumor Necrosis Factor Receptor TNFR1 (DR-1); Fas/CD95 (DR-2); Apoptosis Antigen Apo-3 (DR-3); TNF-related apoptosis inducible ligand-receptor TRAILR (DR-4 and DR-5); Nerve Growth Factor Receptor (NGFR); Ectodysplasin A Receptor (EDAR) that attach their analogous death receptors to the TNF superfamily (TNF alpha; FasL; TL1A; TRAIL; NGF and EDA respectively) and by the expression level of crucial genes elaborated in the extrinsic or intrinsic (perforin/granzyme) apoptotic pathways (Ashkenazi and Dixit, 1998; Wilson et al., 2009). Throughout the progression of apoptosis, the cellular substance has been shown to be retained within the plasma membrane and the apoptotic bodies. As such, the immunogenic cellular components are not secreted into the nearby environment, thus averting an immune-mediated inflammatory response (Bourgine et al., 2013). The lethal-environmental-conditioning method has been utilised by moderating environmental influences, such as temperature and pH, as well as carbon dioxide/oxygen, nitric oxide and hydrogen peroxide content (Somuncu, 2020).

## Assessment of Decellularization Effectiveness

Since 1989, the year in which the pioneer of heart valve surgery Harken DW sanctioned the “ten commandments” for the ideal heart valve substitute (Harken, 1989), none of the developed heart valve replacements has been shown to possess self-repair, adaptively remodelling and growth whilst being resistant to infection and thrombogenicity. However, decellularized porcine tissues (small intestinal submucosa or pulmonary valves) have been utilized for the manufacturing of bioprosthetic heart valves substitutes (Fioretta et al., 2020). The promising results that were reported from preclinical studies did not match those obtained from the clinical application, many of these medical devices, notwithstanding the approval by regulating bodies have been proven to be ineffectively decellularized, eliciting a massive host-specific inflammatory response (Naso et al., 2014). Incidents of leaflets thickening, fibrosis and calcification have been also described. In particular, a strong immunological reaction was reported just a few hours following the implant of the decellularized valve (O’Brien et al., 1999; Dohmen et al., 2003; Leyh et al., 2003; Simon et al., 2003; Dohmen et al., 2005; Ota et al., 2005; Ruiz et al., 2005; Takagi et al., 2006; Iwai et al., 2007; Ruffer et al., 2010; Dohmen et al., 2012; Hopkins et al., 2013; Fallon et al., 2014; Zafar et al., 2015; Hennessy et al., 2017; Mosala et al., 2017; Goecke et al., 2018; Rijswijk et al., 2020).

Preclinical applications of decellularized xenograft valve replacements have been generally issued with the ISO standard for biological medical devices (see Table 3). More recently, a specific standard for the evaluation of decellularized products has become available (ASTM International, 2021). Generally, “decellularization” is a recognized complex process that allows the use of animal-derived ECM products in medical treatment “with reduced risk of adverse host immune response and immune rejection” by disrupting and removing cells and/or cell contents while aiming to preserve significant features of the ECM structure and/or composition (F3354-19, 2021). Unfortunately, the ISO guidelines currently in place do not adequately account for the immunological and pro-inflammatory aspects of such decellularized devices. Within the tissue engineering community, DNA quantification and qualitative evaluation of cell removal have represented common practices for the characterization of the decellularization effectiveness. Residual DNA is responsible for triggering a strong inflammatory response against the implanted decellularized xenogeneic tissue, resulting in calcification and structural degeneration (Simon et al., 2003). A threshold of 50 ng/mg (DNA/tissue) has been commonly accepted to be non-immunogenic (Crapo et al., 2011). Despite this, various studies have reported decellularized porcine small intestine submucosa with up to 6 μg/mg of nucleic acid debris (Naso et al., 2013a; Rijswijk et al., 2020). It is worth noting that often the evaluation of the residual DNA presence after decellularization is only qualitative, limiting the assessment to fluorescence staining based on the use of DAPI (4′,6-diamidino-2-phenylindole, a fluorescent stain that binds strongly to adenine–thymine-rich regions in DNA). Gilbert and colleagues (Gilbert et al., 2009) have proved the existence of DNA fragments in some commercial products even if, when smaller than 300 bp, they do not seem to be enough to stimulate critically the immune system (Soto-Gutierrez et al., 2012).

**TABLE 3:** Common reference ISO Standard for the production of class III implantable biological medical devices.

ISO Identification number	Subtitle	Topics covered
**ISO 13485**	Quality Management System (QMS)	The introduction of MDR (EU) 2017/745 provides that the device manufacturer has a Quality Management System (QMS) to improve and increase efficiency organizational processes
**ISO 14630**	Non-active surgical implants	The standard specifies the general requirements for performance, design, materials, design evaluation, manufacturing, sterilization, packaging and testing to prove compliance with these requirements
	General requirements	
**ISO 14971**	Medical devices - Application of risk management to medical devices	The preparation of the Risk Analysis document allows the identification of hazards connected with the use of a medical device and in quantifying the risk that the damage occurs
**ISO 22442**	Medical devices utilizing animal tissues and their derivatives	This type of devices specific requirement is needed, for example for risk management process, contamination by bacteria, virus or anything else that may cause an undesirable pyrogenic, immunologic or toxicologic reaction
**ISO 14160**	Sterilization of health care products	Liquid chemical sterilizing agents for single-use medical devices utilizing animal tissues and their derivatives
**ISO 10993**	Biological Evaluation on Medical Devices	The standard provides for the realization of a series of tests for the evaluation of biocompatibility of medical devices, concerning their specific type and application
**ISO 11607**	Packaging for terminally sterilized medical devices	The standard provides guidance on how to ensure the sterility of the device once it is entered into the distribution cycle. Validation of the packaging guarantees that the packaging has been made properly for the product it contains
**ISO 14155**	Clinical Investigation of a medical device for human subjects - Good Clinical Practice	The standard provides the technical methodology for designing and carrying out a clinical trial
**ISO 5840**	Cardiovascular implants -- Cardiac valve prostheses Surgically implanted heart valve substitutes	Such a process involving *in vitro*, preclinical *in vivo*, and clinical evaluations are intended to clarify the required procedures before market release and to enable prompt identification and management of any subsequent problems
**IEC 62366**	Application of usability engineering to medical devices	Manufacturers to design for high usability of the product, allowing to limit the risks associated with correct use and errors use of the device

Although quantification of the residual DNA content represents a reasonable way for assessing the effectiveness of decellularization, this approach is limited and not very representative of the immuno-inflammatory risk that can arise following the implantation of decellularized tissues. Homogenized detergent-decellularised scaffolds have been shown to exhibit a high amount of cytosolic and cytoskeleton proteins, such as glyceraldehyde 3-phosphate dehydrogenase (GAPDH) and smooth muscle actin. Interestedly, about 306 proteins of cytosolic, organelle, nuclear and cell membrane origin have been shown to still be identifiable by mass spectrometry (Böer et al., 2011). In addition to the antigenic molecules derived from cell lysis, it is of particular relevance to consider other aspects, such as the remnants of detergents and the presence of pro-inflammatory structures such as hyaluronan fragments.

### Immunocompatibility

Xenograft immunogenicity is directly related to the presence of several antigens including the α-Gal epitope (Naso et al., 2013b), the linked N-glycolyl neuraminic sialic acid (Neu5Gc) (Gao et al., 2017) and the Sd(a) (Byrne et al., 2011). These antigenic molecules are expressed in mammals except for Old World monkeys, apes and humans. Following various stimulation pathways, the human body produces antibodies directed against these molecules. Once a xenogeneic tissue is recognized, the complement cascade is activated, triggering endothelial cell dysfunction, platelet aggregation, and vascular thrombosis (Leventhal et al., 1993; Leventhal et al., 1995; Saadi and Platt, 1995; Hedlund et al., 2008; Pham et al., 2009). In particular, the residual presence of the alpha-Gal xenoantigen significantly increases the human anti-galactose titers specifically directed to the Gala1-3Gal residue, starting from day 10 after BHV implantation and reaching a peak at around 3 months for both IgM and IgG isotypes (Mangold et al., 2009—; Konakci et al., 2005). Such antigenic determinants exposed on the cell surface may persist in membrane residues entrapped between the fibers of the ECM or not properly removed by the action of detergents and may still be capable of reacting despite the elimination of all individual cells (Naso and Gandaglia, 2018).

The treatment most widely used to date to ensure the immunogenic tolerance of animal tissues intended for the manufacture of bioprosthetic heart valves is based on chemical crosslinking with glutaraldehyde (GA). Despite being a benchmark, GA treatment is only partially effective in masking such epitopes (Naso et al., 2013b) resulting susceptible to the development of early BHV degeneration (Hawkins et al., 2016) and immune response following BHV implant (Park et al., 2010—; Schussler et al., 2020). The inability of commercially available treatments capable of effectively inactivating this antigen has recently prompted the U.S. Food and Drug Administration (FDA) to officially approve the production of a genetically modified domestic pig line knocked out for the alpha-Gal in order to provide a source of porcine-based materials for the production of safer and more efficient biomedical devices (U.S. Food and Drug Administration, 2020).

Currently, there are only two technologies that have been reported in the literature to produce complete removal of these xeno-epitopes, including the TriCol decellularization protocol (Gallo et al., 2012) and the FACTA^®^ treatment that has been applied to native and GA-treated animal tissues (Naso et al., 2017).

A correct assessment of the presence of these xeno-epitopes requires an initial *in vitro* evaluation, using suitable antibodies, followed by *in vivo* testing in an animal model. However, current commercially available animal models cannot be considered suitable for this purpose. Wildtype pigs, sheep or rats, like all mammals, do not develop an immunological reaction against these xeno-epitopes, being inefficient in predicting a possible immune-mediated acute or delayed reaction. Owing to this, it is necessary to use animal models that do not express the epitope in question (Old World primates) or humanized animal models, such as genetically modified (knockout) models in which the gene responsible for expression has been inactivated (Smood et al., 2019; Galili and Stone, 2021).

### Detergents Remnants

The decellularization procedures reported in the literature have demonstrated several differences, such as detergent type and concentration, treatment duration, and the number of washes. Most of the studies have indirectly evaluated the amount of detergent residuals in the decellularized scaffolds by determining the amount of the detergents in the washing solutions following cell removal and not directly within the scaffold (Erdbrügger et al., 2006; Caamaño et al., 2009). Effective removal of the detergents is important since the interaction of the ECM with anionic ones has been reported to decrease the tensile strength of elastin fibers (the major component of blood vessels) and to increase the susceptibility of insoluble elastin and collagen to enzymatic degradation (Kagan et al., 1972; Jordan et al., 1974; Guantieri et al., 1983). Elastin degradation products, in turn, can promote the myofibroblastic and osteogenic differentiation of fibroblasts and the *in vivo* recruitment of inflammatory cells (Senior et al., 1980; Simionescu et al., 2007).

Bile acids and salts may be considered natural detergents as they emulsify fats and modify the permeability of cellular membranes (Naso and Gandaglia, 2018). They have been reported to exist in several states in biological systems (even conjugated with sodium, potassium, glycine, taurine, etc.), including insoluble gels, micelles or vesicles, and insoluble calcium salts (Hofmann, 1989). Precipitation of bile acids in the insoluble protonated acid form is mainly controlled by the critical micellization concentration, pH and temperature, Ca^2+^ ion activity, and finally the concentration and structure of the monomeric bile acid anion (Hofmann and Mysels, 1992). Variations of physicochemical conditions may cause the bile salts to transit toward the insoluble gel form, remaining trapped within the ECM fibrillar network determining cell cytotoxicity.

The *in vitro* reseeding of SDS-treated tissues reported controversial results: decellularization of human pericardium and porcine aortic and pulmonary valves using a low concentration of SDS resulted in not cytotoxic drawbacks to *in vitro* cell seeding and cytotoxicity assessment (Mirsadraee et al., 2006—; Vafaee et al., 2018—; Cebotari et al., 2010—; Iop et al., 2014). In a discordant way, Rieder and colleagues reported cytotoxicity of SDS in porcine aortic valves and leaflets treatment (Rieder et al., 2004). In particular, a reduced attachment of human endothelial cells to the matrix was observed (Knight et al., 2005), as well as cytotoxicity of the PBS used for the storage of the decellularized valves, leading to conclude that the residual SDS in the tissue was a cause of cytotoxicity. Noteworthy, even a relatively low residual amount of SDS was correlated with an *in vitro* increased fibroblast activation and *in vivo* foreign body response (Friedrich et al., 2017).

Viability, proliferative capacity and phenotype of human urothelial cells upon urinary bladder matrix scaffolds were maintained on graft treated with Triton X-100, CHAPS and sodium deoxycholate and was similar to cells cultured on the same scaffolds but treated with deionized water (White et al., 2017). SDS treatment resulted in a basement membrane complex with altered ultrastructure and composition, endothelial cells seeded on SDS treated scaffolds reported an atypical morphology, fragmented nuclei and considerably reduced confluence (Faulk et al., 2014).

Reliable information on residual detergents could be used to investigate the relationship, if any, between the actual detergent-based treatment and the observed and/or potential drawbacks in the decellularized scaffold. Additionally, this information could be used for end-product quality control in the manufacturing process of existing decellularised scaffolds currently on the market or under consideration for clinical use. Recently was developed the first cell-based biosensor for residual detergent detection especially in decellularized scaffolds (Ghorbani et al., 2021). This biosensor can be additionally adopted for the evaluation of the cytocompatibility of decellularized tissue giving a comprehensive overview of the general biocompatibility state of the decellularized tissue graft.

### Broken Hyaluronic Acid Molecules

Hyaluronic acid also called hyaluronan (HA), is an anionic, non-sulfated glycosaminoglycan distributed widely throughout connective, epithelial, and neural tissues. HA amounts to 18% in porcine aortic leaflets, 25% in porcine pulmonary leaflets, and 15% in the bovine pericardium (Cigliano et al., 2012). It appears usually as a high-molecular-size polymer of up to 2 × 104 kDa, where disaccharides of N-acetyl-glucosamine and glucuronic acid, connected to each other exclusively by β-linkages, are repeated (Prehm, 1984; Lee and Spicer, 2000). The polymer contributes to the maintenance of tissue hydration (Granger et al., 1984), acting also as an anti-apoptotic agent (Forrester and Balazs, 1980; Jing and DeAngelis, 2004). HA has demonstrated excellent anti-inflammatory and immunosuppressive function due to the ability to coat cell membrane thereby preventing access by the ligand to the surface receptors inhibiting phagocytosis by monocytes, macrophages, and polymorphonuclear neutrophils (PMNs) (Forrester and Balazs, 1980).

During the first step of decellularization, cells lysis may release several types of proteases, some of which will be able to degrade HA (either in free forms or being part of proteoglycans PGs). HA fragments, ranging from 500 Da to 1.5 kDa, have shown a marked pro-inflammatory activity able to activate macrophages with the secretion of various pro-inflammatory chemokines (McKee et al., 1996; Horton et al., 1998). Fragments of 1.35 kDa have been reported to result in inducing dendritic cell maturation, which allowed subsequent priming of allogeneic T-cells to initiate the alloimmunity reaction (Termeer et al., 2000). Specifically, when decellularized tissue is implanted in the human host, the functional effects of HA oligomers and their clearance from the jeopardized tissue stimulate the inflammatory response through their interaction with specific host receptors such as CD44, RHAMM and TLR-2,4 (Rayahin et al., 2015). Lysosomes internalize and further degrade such fragments into tetra and hexasaccharides through the intracellular HYAL1 and HYAL2 (Veiseh and Turley, 2011). These small HA pieces, thus obtained, can act as an endogenous danger signal, leading to the activation of both innate and acquired immunity. The separate stimulation of TLR4 and CD44 receptors may prime/amplify the inflammatory response through NF-kB activation (Loniewski et al., 2007—; Hutás et al., 2008).

It is therefore desirable to ensure that no low-molecular-size HA residues will be available in the decellularized scaffold. When HA fragments are entrapped within the insoluble form of bile salt detergent, or not properly washed out from the ECM, they can transform the decellularised graft into a ticking time bomb, ready to trigger acute immune-mediated reaction once perfused with the recipient’s blood (Naso and Gandaglia 2018).

## Need for the Future

The in-place guidelines contained in the International Organization for Standardization (especially ISO10993 and ISO 5840, Table 3) regulating the manufacturing of decellularized tissue scaffolds appear to need an update. This is evident since the preclinical quantitative assessments of the residual content of xenogeneic reactive molecules, detergents and nucleic acid materials in decellularized scaffolds are insufficient and have led to disastrous results. Decellularized grafts such as the Matrix P prostheses, notwithstanding showed encouraging short-term results, reported unfavourable echocardiographic performances with a relevant dysfunction of the prosthesis in adult patients and a subsequent high rate of reoperation/reintervention for structural pulmonary valve failure (Christ et al., 2019). Histological examination performed by Breitenbach and colleagues exhibited massive inflammatory reaction and necrosis in the Matrix P Plus prostheses adopted as pulmonary homograft in the Ross procedure in adult patients (Breitenbach et al., 2014). Backhoff and colleagues (Backhoff et al., 2014) reported a massive Matrix P^®^ conduit obstruction after 5 years from the implant in 6 years old patients. Conduit dissection allowing blood flow in the thickness of the conduit wall with the formation of pseudoaneurysms was reported, confirming what was already observed also by Rüffer and colleagues (Rüffer et al., 2010). Histological examination showed massive inflammatory reactions and necrosis. Severe inflammation increased fibrous tissue and foreign-body reaction against valve leaflets and fascial tissue was similarly highlighted even by Vogel in young patients (Voges et al., 2013). The reconstruction of aortic heart valves using a decellularized extracellular matrix (CorMatrix^®^) in pediatric patients reported severe valve dysfunction occurred suddenly during the first three post-operative months. A migration of inflammatory cells into the graft edge bordering the native tissue was identified inducing a significant thickening of the patch. The inflammatory reaction induced structural and functional changes (Hofmann et al., 2017).

A strict requirement for any biomedical device is standardization. The consistency and reproducibility of the treatments made according to predefined quality criteria can be ensured, also by adopting new standards capable of adapting to technological progress and new scientific knowledge, such as in the case of knockout animal models. Researchers and the scientific community, while always looking for technological improvement, should converge in the definition of acceptance criteria related to ensuring the consistent evaluation and use of decellularization in manufacturing medical products. Such criteria should address the adequacy of cellular disruption and removal of cellular remnants, defining acceptable quality levels for the remaining extracellular matrix components (Table 4). It would also be appreciable to define a suitable decellularization reagents list placing selective limits on their persistence on the treated ECM.

**TABLE 4:** Summary table showing the approaches commonly adopted for evaluating the efficacy of decellularization processes and the methods of investigation for highlighting potentially dangerous tissue changes according to the future performance of the implant.

Common approaches for the effectiveness evaluation of a decellularization process		
	Approach	Further details
**DNA**	Commercial Kit for DNA extraction and quantification	n.d
	Immunofluorescence assessment (qualitative)	Hoechst, Bisbenzimide H, Draq5, Live red dye, Picogreen
	Histological assessment (qualitative)	Feulgen stain
**Cells**	Immunofluorescence assessment (qualitative)	DAPI (4′,6-diamidino-2-phenylindole)
	Histological assessment (qualitative)	Ematoxylin and Eosin
**Xenoantigens**	Immunohistochemistry assessment (qualitative)	anti-Neu5Gc, anti alpha-Gal and anti-SDa antibodies
	ELISA test (Quantitative)	n.d
**Structural cell proteins**	Immunohistochemistry assessment (qualitative)	anti-alpha-smooth muscle actin, beta-actin, vimentin, collagen VI and laminin antibodies
**Hyaluronan fragments**	Immunofluorescence assessment (qualitative)	Hyaluronan binding protein (HABP) probe biotin-conjugated
**Collagen**	Quantitative evaluation	Hydroxyproline-based assay, sodium dodecyl sulfate-polyacrylamide gel electrophoresis (SDS–PAGE), High-performance liquid chromatography with fluorescence detection (HPLC-FLD)
	Histological assessment (qualitative)	Masson, Mallory and Van Gieson Trichromic staining, Picro Sirius Red
	Immunofluorescence assessment (qualitative)	anti-collagen I, II and III antibodies
**Elastin**	Quantitative evaluation	Desmosine-base assay, NaOH extraction
	Histological assessment (qualitative)	Verhoeff, Orcein, Weigert staining kit
	Immunofluorescence assessment (qualitative)	anti-elastin antibodies
**POST DECELLULARIZATION ASSESSMENT FOR TISSUES PERFORMANCES**		
	**Evaluation approach**	**Commonly considered parameters**
**Mechanical properties**	Bi- or uni-axial stress-strain test for patch-shape tissue	Ultimate Tensile Strength (UTS), Elongation, Young’s Modulus, Compressive Strength (CS), Suture retention Strength (SRS)
	Pulse duplicator assessment for heart valve	Mean Pressure drop (MPD), Effective Orifice Area (EOA), Peak Pressure drop (PPD), Mean Regurgitation and Mean Energy Losses
**Thermal analysis**	Thermogravimetric analysis (TGA)	Protein–water interactions and degradation temperatures
	Differential scanning calorimetry (DSC)	Protein thermal transitions (glass transition and denaturation)
**Detergent residues**	Quantitative chemical evaluation	Dimethylmethylene blue, reverse phase chromatography coupled with UV detection

Similarly, to what happens for the definition and the quality management of any industrial process, a decellularization protocol should also identify according to the step’s objective: 1) the general procedure, 2) materials and equipment utilized, 3) duration or end-point determination, 4) static process parameters, and 5) any variable parameters that may scale with source ECM material characteristics, dimensions, or quantities. Test and inspection procedures should be designed and planned during all the decellularization process, establishing the acceptance criteria in advance. Even if this new standardization approach is unlikely to be applied to products already on the market, it will allow for a more efficient, comparable, and reliable experimental research study to allow a faster technology transfer to the market with an unprecedented level of safety for the patient’s health.
